# Greenhouse gas emissions and energy use associated with production of individual self-selected US diets

**DOI:** 10.1088/1748-9326/aab0ac

**Published:** 2018-03-20

**Authors:** Martin C Heller, Amelia Willits-Smith, Robert Meyer, Gregory A Keoleian, Donald Rose

**Affiliations:** 1 Center for Sustainable Systems, School for Environment and Sustainability, University of Michigan, 440 Church Street, Ann Arbor, MI 48109-1041, United States of America; 2 Department of Global Community Health and Behavioral Sciences, Tulane University, 1440 Canal Street, Suite 2210, New Orleans, LA 70112, United States of America; 3 Author to whom any correspondence should be addressed.; mcheller@umich.edu

**Keywords:** dataFIELD, cumulative energy demand, NHANES, diet shifts

## Abstract

Human food systems are a key contributor to climate change and other environmental concerns. While the environmental impacts of diets have been evaluated at the aggregate level, few studies, and none for the US, have focused on individual self-selected diets. Such work is essential for estimating a distribution of impacts, which, in turn, is key to recommending policies for driving consumer demand towards lower environmental impacts. To estimate the impact of US dietary choices on greenhouse gas emissions (GHGE) and energy demand, we built a food impacts database from an exhaustive review of food life cycle assessment (LCA) studies and linked it to over 6000 as-consumed foods and dishes from 1 day dietary recall data on adults (*N* = 16 800) in the nationally representative 2005–2010 National Health and Nutrition Examination Survey. Food production impacts of US self-selected diets averaged 4.7 kg CO_2_ eq. person^−1^ day^−1^ (95% CI: 4.6–4.8) and 25.2 MJ non-renewable energy demand person^−1^ day^−1^ (95% CI: 24.6–25.8). As has been observed previously, meats and dairy contribute the most to GHGE and energy demand of US diets; however, beverages also emerge in this study as a notable contributor. Although linking impacts to diets required the use of many substitutions for foods with no available LCA studies, such proxy substitutions accounted for only 3% of diet-level GHGE. Variability across LCA studies introduced a ±19% range on the mean diet GHGE, but much of this variability is expected to be due to differences in food production locations and practices that can not currently be traced to individual dietary choices. When ranked by GHGE, diets from the top quintile accounted for 7.9 times the GHGE as those from the bottom quintile of diets. Our analyses highlight the importance of utilizing individual dietary behaviors rather than just population means when considering diet shift scenarios.

## Introduction

Agriculture is a key contributor to many environmental problems, including climate change, biodiversity loss and land and freshwater degradation [1]. Repeated projection studies have shown that closing global yield gaps through sustainable intensification measures will not be sufficient to simultaneously prevent further agricultural expansion and achieve the deep emission cuts needed to meet the COP-21 Paris Agreement on combating climate change. Demand-side reductions will also be necessary [2–5]. Thus, diet composition has been identified as an important leverage point in reducing the environmental impact of food systems and in freeing up production capacity to feed future population growth.

Considerable efforts have been made in recent years to evaluate the environmental impact of dietary choices [6–10]. The bulk of this effort has evaluated aggregated (i.e. average) or stereotyped diets in European countries, with a focus on climate change impacts. Only a handful of studies have evaluated the environmental impact of diets in the US [11–15]. Very few studies, and none in the US, have evaluated the impacts of individual self-selected diets [16–19]. Even the few studies that have attempted this assess only a percentage of foods consumed. Individual-level data are needed for more nuanced modeling of dietary change policies since they allow for understanding the range of impacts within a population and for linking of individual-level demographics (e.g. age, gender, race-ethnicity, education, nutrition knowledge, environmental attitudes, etc.) to the dietary behaviors of these groups and their environmental impacts. Understanding the relation of specific diets to health outcomes also benefits from having the full range of diets chosen by individuals in a population.

A major challenge in this field of research is the establishment of environmental impacts for the diversity of foods in self-selected diets. For example, while a typical study based on aggregated national food patterns might include impacts on a few hundred foods [11], databases that support individual diet surveys contain thousands of items, many of which are complex recipes (e.g. lasagna) or have not been studied in the life cycle assessment (LCA) literature (e.g. blackberry).

Our aim is to evaluate the greenhouse gas emissions (GHGE) and non-renewable cumulative energy demand (CED) associated with a representative sample of self-selected, individual diets in the US. To do this and address the challenge described above, we have developed *dataFIELD* (database of Food Impacts on the Environment for Linking to Diets) based on an exhaustive review of the literature. We have also developed an approach for linking it to dietary recall data from the US National Health and Nutrition Examination Survey (NHANES)^4^.

## Methods

Individual, 1 day dietary recall data (18+ years of age, *N* = 16 800) from NHANES for 2005–2010 serves as the basis for individual food choices studied here. NHANES sampling is selected to represent the US civilian non-institutional population, and the dietary recall data contain reference to 6492 specific foods and dishes [20]. Many of these food items are prepared foods (e.g. pepperoni pizza) and require recipes to assign to the commodity foods typically represented in LCA studies. In order to enable diet-level analysis of pesticide and other residues in food commodities, the US EPA developed the Food Commodities Intake Database (FCID) which links specific food items in NHANES through standardized recipes to foods in agricultural commodity form [21]. We utilize this database to connect as-consumed foods in NHANES to 332 commodity forms, which were in turn connected to environmental impacts based on our literature review.

### Literature review of food LCA data

We conducted a systematic search in Web of Science and Google Scholar databases. Search terms included combinations of ‘LCA’ and ‘life cycle’ with ‘food’. Further refined searches targeted individual underrepresented foods. In addition, collected citations were cross-referenced with the extensive review by Clune *et al* [22] and relevant additional citations were included. The literature review was limited to reports available in the public domain. Articles and reports written in English and published in 2005–2016 that applied LCA methods to one or more food products and provided primary (i.e. not cited from elsewhere) mid-point impact assessment of GHGE and/or CED were reviewed and inventoried. Peer reviewed journal articles as well as thoroughly documented reports from governmental and non-governmental organizations (including theses) were considered. Additional details about our methodology as well as the full listing of references included in our database, *dataFIELD*^5^, is provided in supporting information available at stacks.iop.org/ERL/13/044004/mmedia. For database consistency, mid-point indicator values were adjusted to a functional unit of ‘kg of food,’ with meat and fish/seafood adjusted to ‘kg of edible boneless weight’. See supporting information for details on conversion factors used.

**Table 1.** Process for assigning environmental impact data to FCID commodities.

Stage	Approach for assigning environmental impact data to each specific	Example	% of FCID foods assigned in stage	
	FCID food commodity		GHGE	CED
1	Mean of values from literature review	An average of 96 studies on beef for GHGE, 19 studies for CED		
2	Aggregated value from a report with previously compiled impact data	Kale, from [23]	47%	35%
3	Proxy assignment from stage 1 or 2 foods in the same group OR from stage 1 or 2 foods of similar form	Average of broccoli, cauliflower and cabbage for Brussels sprouts OR bananas for plantains; escarole for radicchio	39%	50%
4	Mass conversion factor applied to base fruit/vegetable	Strawberry values converted for strawberry juice (mass conversion with processing energy added)	15%	15%

### Linking to dietary database

To link environmental impacts to the 332 commodity foods in FCID, we followed a four-step process (see table 1). First, we used data from original research on specific foods inventoried in the literature review, as described above. The mean, standard deviation, minimum and maximum values for CED and GHGE at farm gate and at processor gate were calculated for each specific food, and then matched to the FCID. Studies of heated greenhouse vegetable production or those of beef from dairy herds were not included in our averages because information on market share of these production methods is unavailable or unreliable. Second, if we did not have an original research report on an FCID food, we turned to reports with previously-compiled food LCA data to supply environmental impacts [23–29]. These resources contained data not captured in the literature review, perhaps due to non-English language reports or proprietary sources. Overall, for stage 1 and 2 of the linkage process, CED matches were made for 35% of the food commodities, and GHGE matches for 47%. Third, remaining FCID foods were populated with values from similar foods as proxies. Specifically, we took an average of either CED or GHGE values from existing entries within a specific food grouping (e.g. berries, brassicas, brassica greens, citrus, fresh herbs, grains, other greens, nuts, roots, dried spices, other tree fruit, tropical fruit) to proxy for a specific food item in that same grouping that was lacking data. Failing this approach, other proxies of foods with similar form were then assigned. These assignments were based on similarities of specific crops in their botany and, most importantly, production methods, as determined by the expertise of our research team. Values that were assigned from other foods in the database in this third stage accounted for 50% of CED values and 39% of GHGE values. Fourth, the FCID dataset includes minimally processed forms of fruits and vegetables (e.g. strawberry juice, dried apples). Where direct LCA matches were not available for these forms, we applied a mass conversion factor, gathered from nutritional databases [30, 31], to the base fruit or vegetable in order to approximate the agricultural production burdens of these processed forms. This stage accounted for the remaining 15% of CED and 15% of GHGE values for FCID foods. For juices, vinegar and maple syrup, additional sources were used to develop valid estimates. These additions are detailed in supporting information.

Because of the inconsistency in full life cycle boundary conditions across the literature review entries, cradle-to-farm gate impact factors were chosen for the vast majority of foods. This choice is further supported by the fact that these commodity foods, in many cases, become ingredients in processed, as-consumed foods, and inclusion of life cycle stages downstream from the farm gate would not necessarily reflect impacts of the actual foods consumed. The exceptions to this farm gate boundary condition are foods within the FCID listing that require processing: flours, refined sugars, vegetable oils, etc supporting information contains additional details on these boundary condition choices, as well as an environmentally extended input-output based estimate of the cumulative food processing impacts excluded in this analysis.

### Impact factor variability estimates

Variability is expected in the LCA data gathered for a given food type, both due to differences in agro-climatic conditions and production practices, as well as LCA methodological approaches such as allocation choice. To characterize this variability and estimate its influence on the impacts of diet, we calculated a 95% confidence interval around the average impact for each food, based on the observations for that food (or related foods) that we found in the literature. If there were too few observations for a given food to calculate a confidence interval, we used the confidence interval for a related food or group of foods. We used the lower and upper bounds of this confidence interval in subsequent calculations of diet-level variability. Supporting information also contains details on this method.

**Table 2.** Characterization of literature review and linkage to the FCID, by food group.

Food groups	% of lit. review entries	# of FCID foods^a^	% of FCID foods in group requiring proxy^b^		% of group level impact from proxies^b^	
			GHGE	CED	GHGE	CED
Vegetables	16.8	96	64	72	7.8	18.0
Meats	16.1	10	30	80	0.1	5.6
Beverages	13.4	34	65	68	22.7	10.2
Fruits	12.7	66	55	71	6.2	17.6
Dairy	11.4	3	0	0	0	0
Fish and seafood	9.1	6	0	17	0	9.9
Cereals and grains	6.4	27	52	56	8.0	10.2
Nuts and seeds	4.0	21	48	76	5.2	44.9
Eggs	2.1	1	0	0	0	0
Oils and fats	2.1	13	31	31	0.6	0.4
Legumes	1.8	24	54	67	26.7	59.1
Sweeteners	1.0	9	33	33	42.0	50.1
Other	3.0	22	73	82	4.1	7.3
Total diet	—	332	55	66	2.6	8.2

a Full listing of FCID foods and their impact factors is provided in supporting information. The six processed foods (beer, carbonated drinks, liquor, cheese, yogurt, tofu) not specified in FCID and directly linked to NHANES (i.e. without use of FCID recipe files) in our analysis are included here.

b Includes both proxy levels 3 and 4 (see table 1).

**Table 3.** GHGE and CED of self-selected US diets (age 18+, *n* = 16 800) using average LCA impact factors.

		Consumed		Food loss contributions		Consumed + all losses	
		Mean^a^	SE^a^	Retail losses^b^	Consumer losses^b^	Mean^a^	SE^a^
GHGE (kg CO_2_ eq. per capita)	per day	3.58	0.04	0.25	0.89	4.72	0.05
	per 1000 kcal	1.67	0.01	0.12	0.42	2.21	0.02
CED (MJ per capita)	per day	18.87	0.20	1.41	4.89	25.17	0.30
	per 1000 kcal	8.92	0.07	0.68	2.35	11.95	0.11

a Mean values are calculated using the average impact factor for each food in *dataFIELD*. SE=standard error of the mean, which takes into account variability in diets from one individual to the next, but not variability in the assessments of environmental impacts for a given food. (See figure 1 and accompanying discussion for low and high distributions that do take into account variability in these assessments for each food.) Calculations account for the complex survey design and sampling weights of NHANES.

b Food losses based on USDA’s Loss Adjusted Food Availability dataset (see Methods).

### Linking to NHANES and diet-level calculations

The FCID database contains a recipe file that links foods as reported by NHANES respondents to ingredients in the form of commodities. For example, the recipe for 100 grams of ‘lasagna with meat,’ which is one of 17 lasagna dishes reported by respondents, contains gram quantities of commodities, including wheat flour, milk, beef, tomato, etc. We linked impacts from *dataFIELD* to these FCID commodities and adjusted for recipe quantities and amounts eaten in order to assign impacts for each food consumed during the 24 hour recall day as reported by each respondent. See supporting information for a complete listing of FCID foods and impacts. In some cases, when there was sufficient LCA literature to describe the impact of processed foods (specifically: cheese, yogurt, tofu, beer, carbonated drinks, and liquor) we linked directly from *dataFIELD* to NHANES, without using the FCID recipes. For alcoholic beverages, we created our own recipe file for linking from FCID to NHANES. The impacts of edible losses were calculated for the amount of each commodity consumed using loss factors from the USDA’s Loss-Adjusted Food Availability (LAFA) data series [32]. Commodity items were assigned retail (edible food lost at outlets such as supermarkets and restaurants) and consumer (cooking losses and uneaten food) loss factors from the matching LAFA commodity. If there was not a direct match, the food was assigned loss factors for something similar (e.g. apple juice factors for apricot juice) or for an average of similar items (e.g. an average of loss factors for blueberries, raspberries, and strawberries for huckleberries). After assigning impacts to each food consumed, we summed impacts over the entire day for each individual. All analyses accounted for the NHANES sampling weights and survey design parameters.

## Results

### Literature review characterization

Our comprehensive literature review resulted in 1645 entries (combinations of food types and production scenarios) from 321 unique sources (listed in supporting information). System boundaries varied across the LCA studies inventoried: while nearly all entries considered some form of agricultural production, 51% accounted for processing beyond farm gate, 19% followed products through to retail/regional distribution hubs, and 6% included some form of use (consumption) phase. Supporting information contains additional information on the distribution of entries by publication type and geographic origin of production.

### Food database linkage characterization

Environmental impacts were assigned for the 332 unique food commodity forms in the Food Commodities Intake Database (FCID), and for seven additional foods linked directly to NHANES. Table 2 shows the distribution by literature review entries broken down by broad FCID food groups, with meat, fruit, vegetables and dairy accounting for more than half of the entries. Table 2 also shows the number of FCID foods in each of these groups, as well as the percentage of foods in these groups requiring proxy values. While 55% of foods required proxy in calculating GHGE for the total diet, these foods accounted for only 3% of total impact. This is because the foods requiring proxies tend to be low impact and less frequently consumed foods. For example, the meats group contributed 57% of dietary GHGE (see table 4), but only 0.1% of this group’s impact came from proxies. There were a number of proxies used in the legumes group, accounting for 27% of the GHGE from this group. However, legumes contributed only 0.3% of total dietary GHGE (table 4), so proxied legumes account for only 0.09% of total dietary GHGE.

**Table 4.** Contributions by food groups to impacts of 1 day diets for all diets and for those ranked at the lower and higher quintile by GHGE.

	% contribution to total GHGE^a^			Sum of GHGE per day^a^		
				(metric tons CO_2_ eq. per day)		
	all diets	1st quintile	5th quintile	all diets	1st quintile	5th quintile
Meats	56.6	27.1	70.0	5 95 514	16 458	3 35 141
Dairy	18.3	28.1	11.4	1 92 844	17 066	54 794
Beverages	5.9	11.5	3.7	61 777	6985	17 571
Fish and seafood	5.8	3.4	7.5	60 579	2094	35 826
Eggs	2.8	4.9	1.6	29 815	3009	7469
Vegetables	2.6	5.8	1.5	27 056	3525	7163
Cereals and grains	2.1	5.8	1.1	22 321	3500	5122
Fruits	1.6	4.0	0.9	16 535	2422	4178
Sweeteners	1.4	3.1	0.8	15 064	1903	3864
Other	1.2	2.1	0.7	12 645	1249	3427
Oils and fats	1.0	2.4	0.5	10 306	1464	2564
Nuts and seeds	0.4	0.9	0.2	4154	536	1012
Legumes	0.3	1.0	0.1	3535	617	688
Total of all foods	—	—		10 52 146	60829	478819
Mean caloric intake per capita (kcal per day)	2153	1323	2984			

a Environmental impacts (including retail and consumer losses) for specific foods were summed within each broad food group for each individual (based on NHANES 2005–2010 24 hour diet recall, adults aged 18 and over; *N* = 16 800), and then aggregated across all individuals in the relevant category (total population, 1st quintile, or 5th quintile).

### US diet impact characterization

The NHANES dietary intake survey is representative of the US population. Thus, linking *dataFIELD* to the individual, self-selected diets from NHANES offers a way to estimate the distribution of diet-related impacts across the population on a given day. Table 3 summarizes these results at the distribution mean for the total population on both a per day basis as well as normalized to 1000 kilocalories (kcal) dietary intake. Tables 3 also demonstrates the contribution of food losses to the environmental impact of diet.

**Figure 1.**
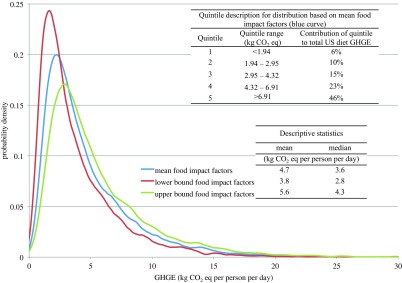
Distribution of diet-related GHGE per person per day among US adults, National Health and Nutrition Examination Survey 2005–2010. Data are based on one 24 hour dietary recall per person and include estimated retail- and consumer-level food losses. Distribution in blue is based on using the mean impact (GHGE/kg food) for each food in our database. Distributions in red and green are based on impact factors (GHGE/kg food) at the lower and upper bounds, respectively, of a 95% confidence interval around these mean estimates of impact for each food.

Figure 1 provides the distribution of diet-related GHGE per person per day across the self-selected diets from NHANES. An analogous figure for CED is included in supporting information. While the distribution in figure 1 shows a sharp rise to a peak in the distribution curve at around 2.2 kg CO_2_ eq person^−1^ day^−1^, there is also a long tail on the distribution (truncated in figure 1: truncated tail represents 1.3% of total impacts). The 20% of diets with the highest carbon footprint account for 45.5% of the total diet-related emissions. Also shown in figure 1 is a range of distributions representing the influence of variability in emissions due to food production methods and LCA modeling.

The distribution of GHGE by food group for all 1 day diets as shown in table 4 is quite typical of Western dietary patterns, with the dominant impacts coming from meats and dairy. An analogous table for CED is included in supporting information. For the total population, 80.6% of the meats group GHGE comes from beef, 9.5% from poultry, 8.5% from pork, with other meats making up the remaining 1.5%. Of interest is the relatively high (5.9% of GHGE, 16.0% of CED) contribution from beverages (tap and bottled water, carbonated drinks, coffee, tea, juices, beer, wine and spirits). Beverages have not always been identified as a separate food group in past diet impact assessments, but were the third most impactful group in our analysis. Across the total population, fruit and vegetable juices make up 33% of the GHGE in the beverages group, followed by coffee, beer, carbonated drinks, cocktails, and wine at 20%, 19%, 9.6%, 8.9% and 7.0%, respectively. Bottled water, tap water and tea contribute less than 2% each.

Table 4 also shows how the contribution by food group differs between lower-impact (1st quintile) and higher-impact (5th quintile) diets. For the higher-impact diets, meats account for 70% of total diet GHGE, whereas they only account for 27% with lower-impact diets. In part, this has to do with the makeup of the meats group in each of the two quintiles. Whereas poultry is the largest contributor in the first quintile (55% of meats group GHGE), beef contributes 91% of meat GHGE in the fifth quintile diets. Although for some food groups the percent contributions in table 4 decline from the 1st to the 5th quintiles (e.g. dairy % contribution to GHGE drops from 28% to 11%), absolute impacts for both GHGE and CED increase for *all* food groups between the first and fifth quintile. This is largely because diets in the 5th quintile have greater amounts of these foods than in the 1st quintile, as suggested by the overall caloric intakes. In terms of overall impact, increases in beef intake account for 72% of the absolute increase in GHGE between the diets of the first and fifth quintile.

GHGE associated with the fifth quintile are 7.9 times those of the first quintile. Total caloric intake is an important factor in ranking impacts per day, as the consumption of more food calories typically translates into greater environmental impact. The fifth quintile consumes, on average, 2.25 times the kilocalories of the first quintile. However, even when impacts are normalized by caloric intake, GHGE of the fifth quintile are five times those of the first quintile. Although this distribution of dietary data from NHANES is representative of diets in the US on any given day, there are a couple of caveats. First, self-reported diets typically understate actual intakes. Second, a distribution of one-day diets is more disperse than a ‘usual intake’ distribution. The low caloric intake reported for the first quintile is, in part, a result of both of these issues. Although we do not have a way to calculate underreporting in this sample, we do know that 26% of respondents in the first quintile reported that their consumption on the recall day was much less than usual.

## Discussion

The *dataFIELD* database described and applied here is an important step in capturing the breadth of food LCA studies in a form that can be linked to existing individual dietary data. It represents one of the more comprehensive compilations available of GHGE and CED data on food production. Further, organizing the database for straightforward linkage with NHANES data creates opportunities for a wide array of future research inquiries, including direct and indirect policy intervention simulations. The sections below provide further discussion on the database development and interpretation of the diet-level results.

### Literature review and *dataFIELD* development

The literature review that underlies development of *dataFIELD* found that LCA studies that can be used to link to dietary choices have increased significantly in recent years, but data gaps still exist for many food types. This is consistent with other recent reviews (e.g. [22]). A scan of the foods requiring proxy assignments in table S4 (supporting information) offers a sense of current data gaps and a target for LCA practitioners interested in filling such gaps. In addition, many foods important to evaluation of healthful diets with low impact—nuts, legumes, meat substitutes—are poorly represented in the literature and deserve additional attention. Geographical representation is biased toward Europe. As has been customary in the diet-LCA literature, our main estimates for diet-level impacts are based on average LCA values applied to each food consumed. However, unlike other studies, we have addressed variability due to production practice, geography, or LCA method by calculating upper and lower bounds of impacts for each food and carrying these estimates through to diet-level impacts. As the NHANES dietary recall data does not specify production methods or geographical origin, we cannot be more precise in assigning impacts from LCA studies to foods eaten by NHANES respondents. However, geographical specificity becomes increasingly important with other impact categories such as water use, eutrophication, or land use. Although currently available data in these categories are limited, we plan to expand our database to water and land use impacts, specific to the US food market, in a future iteration.

**Figure 2.**
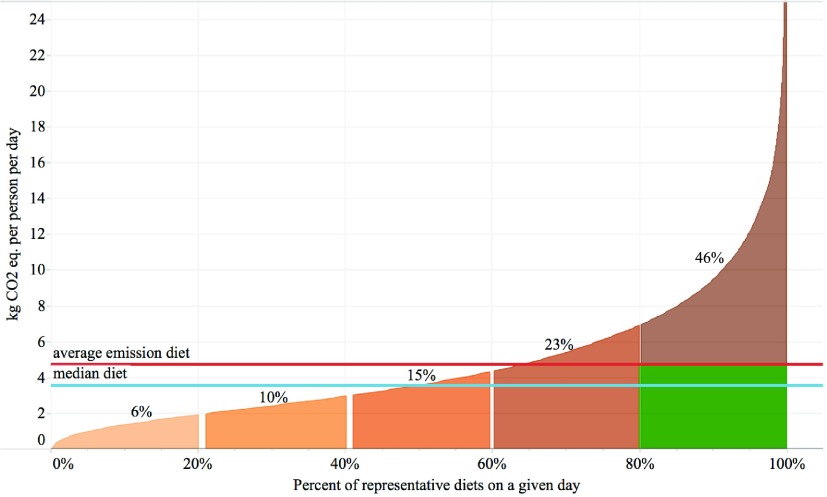
Cumulative emission intensity of US 1 day diets using average impact factors. Diets are ranked in order of impact from low to high. Areas under the curve are proportional to the total impact, with percentage contributions by each quintile shown above the curve. The green box represents the cumulative emissions of those originally in the 5th quintile if their diets were to shift to diets with average emission intensities.

It has become common practice in diet impact studies to assign proxy foods as approximations in the case of missing data, but to our knowledge, this paper is the first attempt to quantify the contributions from those proxy assignments. Table 2 indicates that proxy foods contribute 3% to the average diet GHGE, and 8% to CED. Proxy assignments are made based on foods with similar production characteristics. However, even if we assume that *all* of our proxy estimates are in error by a factor of 2 (i.e. all proxy impact factors are doubled), the mean diet-level impacts would still only increase by 2.6% for GHGE and 8.2% for CED.

### Diet-level impacts: interpretation and comparison with previous results

This study demonstrates the disproportionate impacts that can be caused by some types of self-selected diets. Figure 2 displays the cumulative emissions of these diets when ranked in order of GHGE per person per day. GHGE associated with the fifth quintile of diets are nearly eight times that of the first quintile and three times that of the third (middle) quintile. If the top quintile of diets (representing 44.6 million Americans on a given day^6^) shifted such that their associated GHGE were aligned with the mean impact, this would represent a one-day reduction in GHGE of 0.27 million metric tons CO_2_ eq. (mmt), equivalent to eliminating 661 million average passenger vehicle miles^7^ on a given day.

**Table 5.** Comparison of studies estimating impacts of the US diet or self selected diets in other countries.

	Country	Diet data source^a^	Impact factor data source	GHGE kg CO_2_e capita^−1^ day^−1^		CED MJ capita^−1^ day^−1^
				consumed	consumed+losses	consumed+losses
This study	US	NHANES national survey (SS)	Exhaustive lit. review	3.6	4.7	25.2
Heller and Keoleian 2015 [11]	US	USDA (FB)	limited lit. review	3.6	5.0	
Tom *et al* 2016 [12]	US	USDA (FB)	[11], lit. review		5.1	34.5
Hallstrom *et al* 2017 [15]	US	USDA (FB)	Lit. review	3.8		
Vieux *et al* 2012 [17]	France	INCA2 national survey (SS)	Lit. review	4.2		
Meier and Christen 2013[19]	Germany	German National Nutrition Surveys (SS)	Hybrid EIO LCA		5.6	37.0
Rugani *et al* 2013 [43]	UK	National Diet and Nutrition Survey (SS) + FB to estimate waste	Lit. and other (cradle to point of sale)		8.8^b^	
Van Dooren *et al* 2014 [53]	Netherlands	Dutch National Food Consumption Survey (SS)	Agri-footprint data [23]	4.1		
Hendrie *et al* 2016 [54]	Australia	Australian Health Survey (SS)	EIO LCA	18.7^b^ (male) 13.7^b^ (female)		
Bälter *et al* 2017 [44]	Sweden	LifeGene study (SS)	Lit. identified sources		4.7	

a (SS) = self-selected diet; (FB) = food balance.

b Represents broader boundary conditions than other studies; includes impacts through to the point of purchase.

This shift—which could be done by changing foods, reducing calories, or some combination of these two—would be represented graphically in figure 2 by removing the section of the curve above the average emission diet line for the fifth quintile. Current economy-wide US net emissions (based on 2015 data [34]) are 1023 mmt above the target levels in year 2025, as submitted to the U.N. Framework Convention on Climate Change (UNFCCC) [35]. The hypothetical diet shift described above, if implemented every day of the year and met by equivalent shifts in domestic production, would account for 9.6% of remaining reductions necessary to meet the target. (see supporting information for the emission reduction calculations.) Even if high emission diets (arbitrarily defined here as >25 kg CO_2_ eq. person^−1^ day^−1^; the truncated tail extending above the representation in figure 2) are excluded from the estimate based on a presumption that they are either atypical or that such individuals are unlikely to shift diets, moving the remainder of the high quintile (GHGE >6.9 but <25 kg CO_2_ eq. person^−1^ day^−1^) to the mean GHGE still accomplishes 9% of the reductions necessary for the US to meet the UNFCCC target. See supporting information for a parallel discussion on the cumulative impacts of food losses. Our estimates of reductions are likely to be somewhat exaggerated because a distribution of 1 day diets is known to be more dispersed than a distribution of usual diets [36]. This is one of the limitations of using NHANES. Since it is based on the 24 hour diet recall tool, it also tends to underestimate total energy intake, although this is true of all self-reported diet instruments [37]. In fact, 24 hour recalls provide more details about foods consumed and tend to be less biased than food frequency questionnaires [38]. Moreover, NHANES provides the only ongoing nationally representative source for information about individuals’ diets. Our analysis highlights the importance of looking at individual behaviors rather than just population means, since there is clearly a wide range of impacts being caused by self-selected diets.

Table 5 offers a comparison of the results from this study with other reported estimates of the impacts of the US diet, as well as self-selected diets in other countries. When excluding studies that include broader boundary conditions, there is strong agreement across results, with a coefficient of variation of 3% for GHGE with US diets only, and 7% across all diets. Self-reported diet surveys carry a well-known under-reporting bias [39, 40] whereas food balance based estimates (production + imports—exports—non-food uses ± changes in stock) are often considered to be overestimates [41, 42]. A more refined food type characterization and a more exhaustive literature review were utilized in this study in comparison to that of Heller and Keoleian [11]. While beverages have not always been delineated as such in previous studies of diet impacts [11, 12, 15, 19, 43–44], we find them to be important contributors. This finding is further strengthened by the fact that packaging and use phases are *not* included within the boundary conditions of our estimates. Packaging often represents a hotspot in the life cycle impacts of beverages [45–49], and use phase activities (heating water, brewing coffee) can be important for hot beverages [50, 51].

The boundary conditions for the current study are cradle to farm gate for most food commodities, and include processing for the collection of FCID foods that are minimally processed ingredients (flours, oils, juices, etc). As such, our reported values should be considered underestimates of actual impacts associated with food consumption in the US as they include the production impacts of processed food ingredients, but not the impacts of processing itself. Using the US Environmentally Extended Input Output model developed by US EPA [52] and an approach detailed in supporting information, we estimate that food processing not captured in our bottom-up estimates amounts to 15% of the total cradle to processor gate (including agricultural production sectors) GHGE. Packaging materials represent an additional 6%. Inclusion of these missing food processing and packaging contributions would raise our estimates by ~27%, although it is important to note that these input-output based approximations are made for the food and agricultural sectors in aggregate, and will not apply evenly across different food types or for specific diets (i.e. they apply only at the mean).

### Impact factor variability

In figure 1, we demonstrate the influence that food impact factor variability, as represented by our literature review, has on the diet-level impacts of the US population. Based on this estimated variability, the GHGE for the mean of the population ranges from 3.8–5.6 kg CO_2_ eq. person^−1^ day^−1^, or ±19% of the value based on average impact factors. Variability of food production systems across geographies and production methods is expected. In most cases, the granularity of available LCA data is not sufficient to reasonably and consistently differentiate between these food production variables. On top of this, methodological choices within LCAs, such as how impacts are allocated between co-products, introduce an additional level of variability between studies that cannot be effectively disaggregated from production variability. It is important to keep in mind, however, that even if such environmental impact data were complete, the corresponding information in diet databases is not available. NHANES represents the best information on diet—both in the aggregate and in its diversity across the population—available for the US Yet, it does not (currently) contain information on the methods of production for food sources (e.g. was a tomato grown in a heated greenhouse? Was it organically grown?) or geographic origin of production (California? Michigan? Chile?). To further refine these estimates would require more information on foods in the NHANES survey, as well as better LCA data on food production variability.

## Conclusion

This paper describes the development of *dataFIELD,* a food production environmental impact database based on an exhaustive review of the LCA literature, and provides a framework for linking this data with individual self-selected diets of the US population. The study demonstrates the distribution of diet-related GHG and energy demand intensity for self-selected diets in the US, showing that the fifth of the diets with the highest carbon footprint account for 46% of the total diet-related GHGE burden. Behavior change campaigns focused on these diet types could be an efficient and effective means of reducing US GHGE. Campaigns targeting dietary shifts, therefore, offer a significant opportunity for state, city, business and other organizational policy or leadership aimed at climate change action. Getting people to change dietary behavior is notoriously challenging [55], and enhanced efforts are needed to better identify effective strategies for influencing diet shifts that lead to reduced environmental impacts.

Data gaps are often a major challenge in LCA. This study demonstrates for the first time, however, that foods for which no LCA data currently exist do not represent a significant contribution to the carbon intensity of US diets. Further, we quantify the influence of variability in LCA data on impacts at the diet level to be ±19% of the mean. While current diet recall data do not capture the information necessary to do so, future work could connect food choice and diet variation of individuals with a more precise characterization of the supply chains producing their food to better understand the implications of sourcing. Given the ranges in production impacts across practices and geographies, this may also be an important aspect in reducing food system impacts.

Future work will investigate correlations between environmental impacts and health implications of individual US diets, as well as elucidate associations between population demographics and diet-related environmental impacts. Combined, these works provide a solid foundation for policy considerations that acknowledge diet shifts as an instrumental component of GHGE reduction goals.

## Footnotes

4 NHANES dataset with GHGE per person for day one diets and accompanying documentation will be available at: https://sph.tulane.edu/gchb/diet-environmental-impacts.

5 The complete dataFIELD database is available at: http://css.umich.edu/page/datafield.

6 The total population number utilized is the represented population for the 2005–2010 NHANES data, denoting the civilian, non-institutionalized, age 18+ population at the midpoint of the time period: 222 909 266.

7 Based on a US weighted average combined fuel economy of cars and light trucks of 9.35 kilometers (km) liter^−1^ (22.0 miles per gallon) [33].
